# Supplementary material for: Decreased Vitamin D Levels and Altered Placental Vitamin D Gene Expression at High Altitude: Role of Genetic Ancestry
Source: Int J Mol Sci. 2023 Feb 8;24(4):3389. doi: 10.3390/ijms24043389 (PMC9967090; doi:10.3390/ijms24043389)
Supplement: Supplementary file 1 [file ijms-24-03389-s001.zip › ijms-2102958-supplementary.pdf]

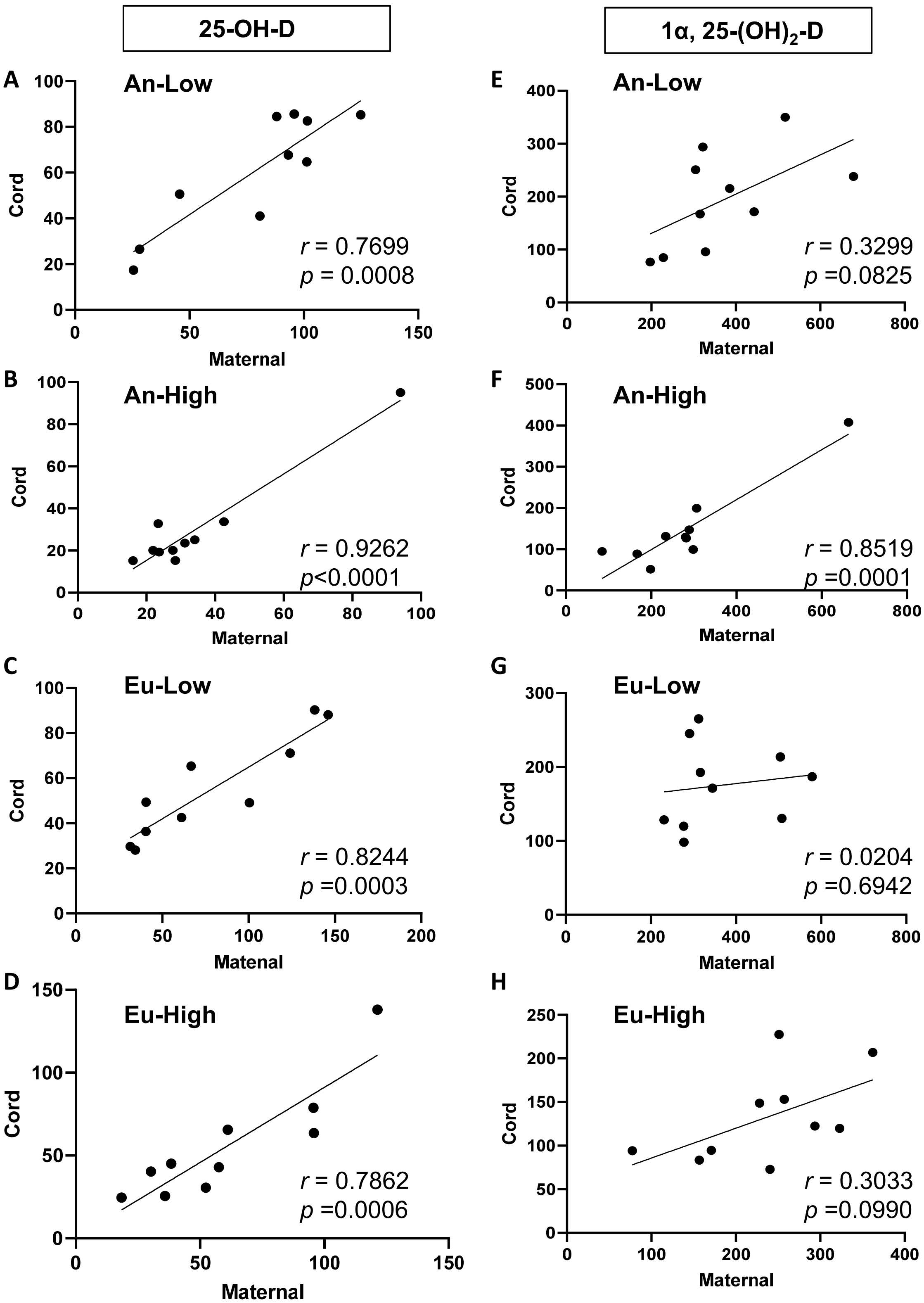

Supplementary Figure S1. Correlations between maternal and umbilical cord blood levels of vitamin D metabolites. A-D) linear regression analysis between maternal and fetal/neonatal levels of 25-OH-D (nanomoles/L) in An-Low (A), An-High (B), Eu-Low (C), and Eu-High (D). E-G) linear regression analysis of 1α,25-(OH)$_2$-D (picomoles/L) levels correlation between maternal and fetal/neonatal samples in An-Low (E), An-High (F), Eu-Low (G), and Eu-High (H). Insets show the correlation coefficient and *p* value.

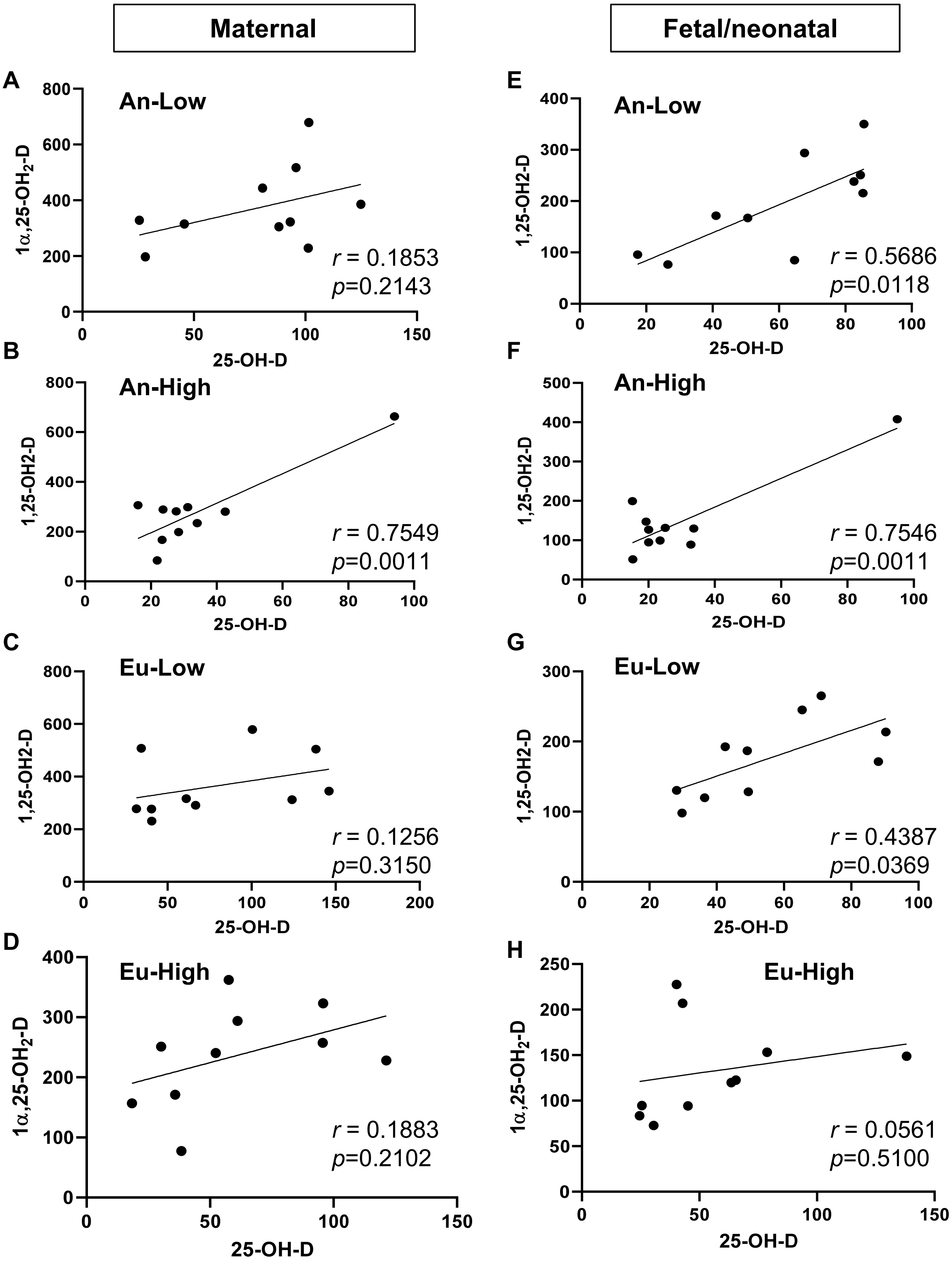

Supplementary Figure S2. Correlations between 25-OH-D and 1α,25-$(OH)_2$-D. Linear regression analysis of the precursor 25-OH-D (nanomles/L) and the bioactive form 1α,25-$(OH)_2$-D (picomoles/L) is shown for maternal blood of An-Low (A), An-High (B), Eu-Low (C), Eu-High (D) as well as for umbilical cord blood (fetal/neonatal) samples of An-Low (E), An-High (F), Eu-Low (G) and Eu-High (H) groups. Insets show the correlation coefficient and *p* value.
